# HIF-1α Signaling Activation by Post-Ischemia Treatment with Astragaloside IV Attenuates Myocardial Ischemia-Reperfusion Injury

**DOI:** 10.1371/journal.pone.0107832

**Published:** 2014-09-19

**Authors:** Jingwen Si, Ning Wang, Huan Wang, Juan Xie, Jian Yang, Hui Yi, Zixuan Shi, Jing Ma, Wen Wang, Lifang Yang, Shiqiang Yu, Junchang Li

**Affiliations:** 1 Department of Traditional Chinese Medicine, Xijing Hospital, The Fourth Military Medical University, Xi'an City, Shaanxi Province, China; 2 Department of Cardiovascular Surgery, Xijing Hospital, The Fourth Military Medical University, Xi'an City, Shaanxi Province, China; 3 Department of Dermatology, Tangdu Hospital, The Fourth Military Medical University, Xi'an City, Shaanxi Province, China; 4 Department of Nephropathy and Immunology, BaYi Childrens Hospital of The General Military Hospital of Beijing PLA, Beijing, China; 5 Department of Anesthesiology, Xijing Hospital, The Fourth Military Medical University, Xi'an City, Shaanxi Province, China; Indiana University School of Medicine, United States of America

## Abstract

In this study, we evaluated the effect of astragaloside IV (Ast IV) post-ischemia treatment on myocardial ischemia-reperfusion (IR) injury (IRI). We also examined whether hypoxia inducible factor-1α (HIF-1α) and its downstream gene-inducible nitric oxide (NO) synthase (iNOS) play roles in the cardioprotective effect of Ast IV. Cultured cardiomyocytes and perfused isolated rat hearts were exposed to Ast IV during reperfusion in the presence or absence of the HIF-1α inhibitor 2-methoxyestradiol (2-MeOE2). The post-ischemia treatment with Ast IV protected cardiomyocytes from the apoptosis and death induced by simulated IRI (SIRI). Additionally, in cardiomyocytes, 2-MeOE2 and HIF-1α siRNA treatment each not only abolished the anti-apoptotic effect of post-ischemia treatment with Ast IV but also reversed the upregulation of HIF-1α and iNOS expression. Furthermore, after treatment with Ast IV, post-ischemic cardiac functional recovery and lactate dehydrogenase (LDH) release in the coronary flow (CF) were improved, and the myocardial infarct size was decreased. Moreover, the number of apoptotic cells was reduced, and the upregulation of the anti-apoptotic protein Bcl2 and downregulation of the pro-apoptotic protein Caspase3 were reversed. 2-MeOE2 reversed these effects of Ast IV on IR-injured hearts. These results suggest that post-ischemia treatment with Ast IV can attenuate IRI by upregulating HIF-1α expression, which transmits a survival signal to the myocardium.

## Introduction

Ischemia and reperfusion (IR) injury (IRI) is a primary cause of cardiac failure, morbidity, mortality after cardiac operations [1] or heart infarctions [2]. Determining how to salvage the viable myocardial tissue and restore its electrical and mechanical functions has become a primary focus in clinical settings [3]. There are many powerful strategies to limit IRI [4], [5], [6]. However, many strategies involve procedures with certain limitations (such as safety and ethics). Thus, alternative methods have been explored, including protective drug delivery at the beginning of reperfusion [7], [8].

**Figure 1:**
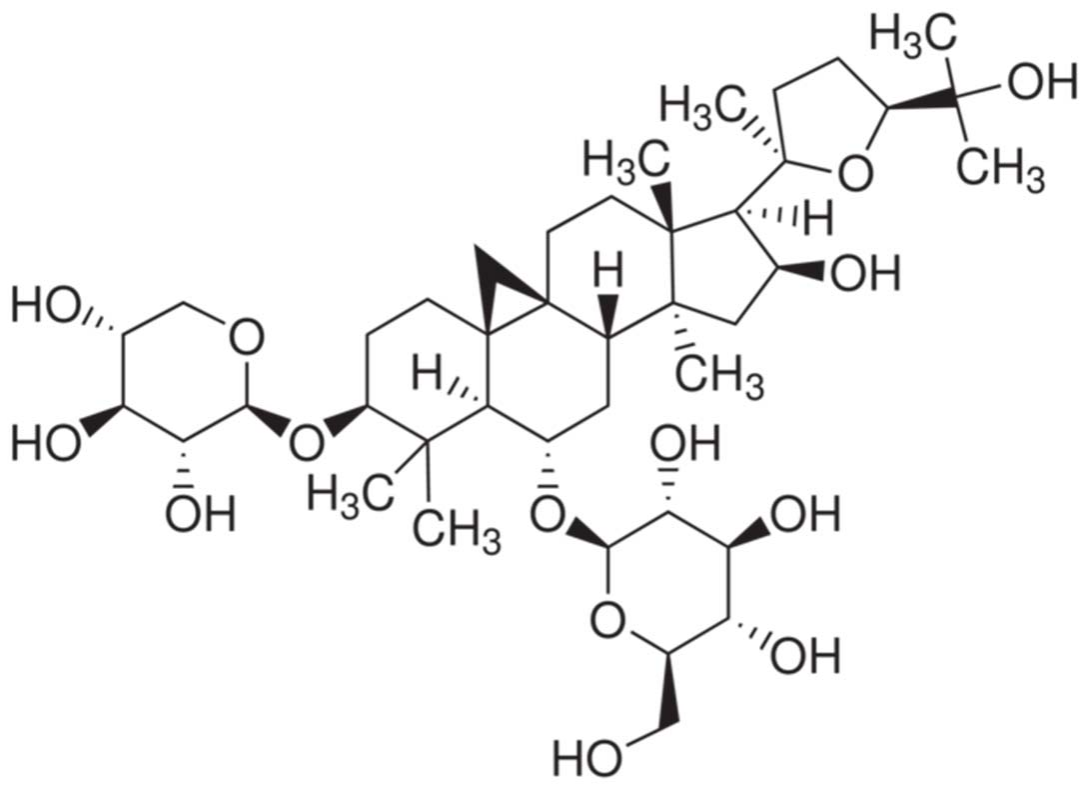
The chemical structure of Astragaloside IV.

Astragalus membranaceus, which is a Chinese traditional medicine, has long been used for the management of various diseases [9]–[17]. Medicinally active compounds have been isolated from this plant, including astragalosides, polysaccharides, and flavones. Astragaloside IV (Ast IV, which has the chemical structure shown in Figure 1) is one of the main active constituents of astragalosides. Ast IV is non-toxic and non-mutagenic, and it mediates a wide spectrum of biological functions, such as cardioprotection, metabolic syndrome, antioxidant and anti-carcinogenic properties [9]. Recent reports have indicated that Ast IV can attenuate IRI in the brain [10], kidney [11], liver [12], retina [13], and skin [14] under various experimental conditions. The protective effects against myocardial IRI have been reported in different animal models in which Ast IV was administered before ischemia (Ast IV pre-treatment) [15]–[17]. However, whether post-ischemia treatment with Ast IV has a potential protective effect against IRI in the heart has not been well investigated.

Over the past decade, the transcriptional complex hypoxia inducible factor-1α (HIF-1α) has emerged as a key regulator of the molecular hypoxic response and is a master regulator of the cellular and systemic homeostatic responses to hypoxia by activating the transcription of many genes, including those involved in energy metabolism, angiogenesis, apoptosis, and other genes, the protein products of which increase oxygen delivery or facilitate metabolic adaptation to hypoxia [18]. HIF-1α plays an essential role in embryonic vascularization, tumor angiogenesis, and the pathophysiology of ischemic disease. In particularly, HIF-1α activation plays an essential role in triggering cellular protection and metabolic alterations in response to oxygen deprivation during myocardial ischemia [19]. It has been reported that an increase in the level of HIF-1α is one of the first adaptive responses that occur at the molecular level of the myocardium to ischemia [20]. Experimental studies have suggested that HIF-1α may act as a mediator of ischemic preconditioning and that the genetic or pharmacological stabilization of HIF-1α under normoxic conditions may protect the heart against the detrimental effects of acute IRI [21]. Other studies have demonstrated that post-conditioning reduces infarct size, attenuates apoptosis, and up-regulates the expression of HIF-1α [22], [23]. It has also been reported that Ast IV stimulates angiogenesis and increases HIF-1α accumulation via the phosphatidylinositol 3-kinase/protein kinase B (PI3K/Akt) pathway [24]. However, whether HIF-1α plays an important role in the cardioprotection of post-ischemia treatment with Ast IV requires further investigation.

HIF-1α attribute to the activation of Inducible nitric oxide synthase (iNOS), which in turn further stabilize HIF-1α and rapidly amplify the innate defense pathway [25]. Regarding the mechanisms of iNOS-mediated protection, several recent studies have reported that NO generated from activated iNOS stimulates guanylate cyclase enhances the formation of cGMP and thereby activates protein kinase G, leading to the subsequent opening of mitochondrial ATP-sensitive K^+^ channels and the inhibition of the mitochondrial permeability transition pores [26]–[28]. It has also been previously reported that NO donors under normoxic conditions stimulate HIF-1α expression, resulting in iNOS expression and NO generation [29]. By virtue of these results, HIF-1α has become an attractive molecular target for limiting IRI through modulating iNOS gene expression.

In this study, post-ischemia treatment with Ast IV was used as an adjuvant to foster the amelioration of IRI in cultured cardiomyocytes and isolated perfused rat hearts, and the involvement of the HIF-1α signaling pathway in the protection was also evaluated.

## Materials and Methods

### Materials

This study was performed according to the Guide for the Care and Use of Laboratory Animals, which was published by the U.S. National Institutes of Health (National Institutes of Health Publication No. 85–23, revised 1996), and was approved by the Ethics Committee of the Fourth Military Medical University. All experiments were performed on healthy adult male Sprague-Dawley rats that weighed between 220–250 g or on 1- to 2-day-old Sprague-Dawley rats, which were obtained from the animal center of the Fourth Military Medical University. The rats were kept in a pathogen-free environment with free access to food and water.

Ast IV, 2-Methoxyestradiol (2-MeOE2), collagenaseI, bromodeoxyuridine (BRDU), triphenyltetrazolium chloride (TTC), 5-diphenyltetrazolium bromide (MTT), dimethyl sulfoxide (DMSO), ethylene diamine tetraacetic acid (EDTA), and 4′6-diamino-2-phenylindole (DAPI) were purchased from Sigma-Aldrich (St. Louis, MO, USA). HIF-1α siRNA, siRNA transfection reagents, HIF-1α antibody, and iNOS antibody were purchased from Santa Cruz Biotechnology (Santa Cruz, CA, USA). Bcl2, Caspase3, and β-actinantibodies were purchased from Cell Signaling Technology (Beverly, MA, USA). Terminal deoxynucleotidyl transferase dUTP nick-end labeling (TUNEL) kits were purchased from Roche (Mannheim, Germany). Dulbecco's modified eagle medium (DMEM) and fetal calf serum were purchased from Hyclone Company (Logan, Utah, USA). The fluorescein isothiocyanate (FITC)-Annexin V/propidium iodide (PI) staining kit and Bradford protein assay kit were purchased from the Beyotime Institute of Biotechnology (Nanjing, Jiangsu, China). The Bradford protein assay kit was purchased from Pierce Company (Rockford, IL USA). Rabbit anti-goat, goat anti-rabbit, and goat anti-mouse secondary antibodies were purchased from Zhongshan Company (Beijing, China).

### Preparation of primary neonatal cardiomyocytes and simulated ischemia reperfusion (SIR) treatment

The primary cultures of neonatal rat cardiomyocytes from 1- to 2-day-old Sprague-Dawley rats were prepared using a previously reported method [30]. Newborn Wistar rats were disinfected with 70% ethanol and then euthanized by cervical dislocation. Hearts were excised and placed in a sterile ADS solution (116 mM NaCl, 20 mM HEPES, 80 µM Na_2_HPO_4_, 56 mM glucose, 5.4 mM KCl, 800 mM MgSO_4_–7H_2_O; pH 7.35). Blood and connective tissue were removed, ventricles were minced and subjected to five and 15 min enzymatic digestions using collagenase I and pancreatin. Rat neonatal cardiac fibroblasts (RNCF) and myocytes (RNCM) were separated via pre-plating for 2 h. Following isolation, RNCM were cultured overnight in DMEM media containing 10% FBS and 1% BRDU at 37°C in a humidified incubator with 5% CO_2_. The following day, media were replaced. 3–4 days later, the cells were used for the further experiments.

The SIR was performed using concentrations of potassium, hydrogen, and lactate, similar to those that occur in vivo. Briefly, the cardiomyocytes were exposed to an ischemic buffer containing the following reagents (in mM): NaCl (137), KCl (12), MgCl_2_ (0.49), CaCl_2_·2H_2_O (0.9) and HEPES (4) [31]–[33]. This buffer was also supplemented with the following compounds (in mM): deoxyglucose (10), sodium dithionate (0.75) and lactate (20). The buffer pH was 6.5, and the cells were incubated for 2 h in a humidified cell culture incubator (21% oxygen, 5%CO_2_, 37°C). Reperfusion was initiated by returnning to a normal humidified cell culture incubator (21% oxygen, 5%CO_2_, 37°C) and normal medium for 4 h. At the onset of reperfusion, the cardiomyocytes were randomly exposed to one of the following treatments: normal culture medium (DMEM media containing 10% FBS and 1% BRDU), Ast IV (0, 12.5, 25, 50, 100, or 200 µM) with or without 2-MeOE2 (5 µM, based on preliminary experiments) in culture medium.

### Cell viability

Cell viability was assessed using the MTT assay. After treatment, the cells were washed with PBS, and 100 µL of a 0.5 mg/ml MTT solution in phenol red-free DMEM was added to the cell cultures. The samples were incubated for 4 h at 37°C. Next, 100 µL of DMSO was added to each well, and the samples were shaken for 15 min at 37°C. The absorbances were measured at 490 nm ona SpectraMax 190 spectrophotometer (Molecular Device, Sunnyvale, CA, USA). The reduction in optical density (OD) was considered to be the decrease in the survival rates.

### Flow cytometry to measure apoptosis

Cell apoptosis was detected with the FITC-Annexin V/PI staining kit. After treatment, the cells were harvested, washed in ice-cold PBS, incubated for 15 min with fluorescein-conjugated Annexin V and PI, and analyzed on a FACScan flow cytometer equipped with the FACStation data management system and Cell Quest software (all from Becton Dickinson, San Jose, CA, USA).

### Isolated perfused rat heart preparation

As described previously [34], SD rats were intraperitoneally anesthetized with sodium pentobarbital (50 mg/kg). Twenty minutes after an intraperitoneal injection of 500 U/kg heparin sodium, the chest was opened. The heart was quickly removed and retrogradely perfused through the aorta with a non-circulating Langendorff apparatus (Radnoti Glass Technology Inc., Monrovia, CA, USA) at a constant pressure of 80 mmHg. The perfusate was Krebs-Henseleit buffer (KHB) containing (in mM) 118 NaCl, 4.7 KCl, 1.2 MgSO_4_, 1.2 KH_2_PO_4_, 1.25 CaCl_2_, 25 NaHCO_3_, and 11 glucose (pH 7.4, 37°C, which was constantly gassed with 95% O_2_ and 5% CO_2_. The left ventricular developed pressure (LVDP) and heart rate (HR) were monitored via a transducer (Model 100 BP-Biopac System Inc., Goleta, CA, USA) that was connected to a water-filled latex balloon inserted into the left ventricle through the left atrium. At the beginning of the experiment, the left ventricular end-diastolic pressure (LVEDP) was adjusted to approximately 5 mmHg by inflating the balloon. The isolated heart was surrounded by a homoeothermic glass cover (37°C) to maintain temperature. All the of the data were recorded and stored with the AcqKnowledge 3.8.1 software package and a Biopac Data Acquisition System (Model 100 BP-Biopac System Inc.). The index of the myocardial function was determined by the LVDP and the maximum rate of change in pressure development (+dP/dt max). The LVDP is presented as the difference between the left ventricular peak systolic pressure and the end-diastolic pressure, and the +dP/dt max is the rate of change in pressure development, which is also a typical indicator of cardiac function. The coronary effluent was collected during the reperfusion for biochemical assays. Ast IV (20 µM, based on preliminary experiments) with or without 2-MeOE2 (2 µM, based on preliminary experiments) was added to the KHB during the first 10 min of reperfusion.

### Quantitation of myocardial infarct size

At the end of the reperfusion period, each heart was rapidly excised and serially sectioned length-wise into six slices, followed by incubation in 1 % TTC for 20 min at 37°C to demarcate the viable and non-viable myocardium. The isolated hearts were subjected to global ischemia; therefore, the entire ventricle was considered to be area at risk (AAR). The normalized infarct size was expressed as the ratio of the infarct size to the total AAR. An observer (blind protocol) was assigned to assess the percentage of infarcted area using computer-assisted planimetry technique (OPTIMAS v. 5.2, BioScan Inc., Edmonds, WA, USA) [30].

### Measurement of lactate dehydrogenase (LDH) release into the culture medium and coronary effluent

Cardiomyocyte injury was assessed by measuring LDH release into the culture medium. Briefly, at the end of treatment, the incubation medium was stored at 4°C, and the same volume of cold buffer (10 mM Tris-HCl, pH 7.4, 1 mM EDTA) was added to the cells. The cells were then scraped and lysed by trituration. The lysates were centrifuged at 4°C, and the supernatant was stored at 4°C. The LDH concentrations in the medium (released LDH) and in the cell lysate (retained LDH) were measured using a spectrophotometric assay. The results are expressed as the percentage of released LDH relative to the total (released plus retained) LDH. Isolated heart injury was assessed by measuring the LDH concentration in the coronary effluent. The LDH levels in the coronary effluent were determined using the same ELISA kit, following the manufacturer's instructions. The amount of LDH released during the 60 min of reperfusion was determined by by calculating the total amount of LDH protein from individual 5-min collection of coronary effluent. The LDH level was normalized against the dry heart weight and expressed as IU/g. And the lyophilized tissue was dried in an oven at 100°C until its dry weight became constant.

### Quantitation of apoptotic cardiomyocytes

A portion of the myocardium from the mid-left ventricle of the middle slices was fixed in 4% formalin. The level of myocardial apoptosis was analyzed by TUNEL staining. A double-staining technique was used; TUNEL staining was used to quantitate apoptotic cell nuclei, and DAPI staining was used to quantitate the total myocardial cell nuclei. The TUNEL-positive cells that showed green nuclear staining and all of the cells with blue nuclear DAPI staining were counted within five randomly chosen fields under a high power magnification. The index of apoptosis was expressed as the ratio of positively stained apoptotic myocytes to the total number of myocytes counted×100%.

### Real Time Reverse Transcription–Polymerase Chain Reaction (Real time RT-PCR) Measurements of mRNA

Real-time RT-PCR was used to assess abundances of iNOS and β-actin mRNA. Total RNA was isolated using Trizol Reagent (Invitrogen, Carlsbad, CA, USA). cDNA was synthesized from total RNA with Taqman reverse transcriptase (Applied Biosystems, Foster City, CA, USA). cDNA were amplified in a Smart Cycler II (Cepheid, Sunnyvale, CA, USA) by a SYBR Green Polymerase Chain Reaction Master Mix (Promega, Madison, WI, USA) and an Applied Biosystems 7500 Real-Time Polymerase Chain Reaction System (Carlsbad, CA, USA). Two-step real-time polymerase chain reaction was performed (95°C for 15 seconds, 60°C for 60 sec extension and detection, 40 cycles) with specific primers for iNOS (forward 5′-CTTTTAGAGACGCTTCTGA G-3′ and reverse 5′-TTTGATGCTTGTGACTCTTA-3′) and β-actin (forward: 5′-TCTTTTCCAG CCTTCCTTCTTG-3′; reverse: 5′-GCACTGTGTTGGCATAGAGGTC-3′). Abundances of amplified genes were assessed by analysis of cycle threshold.

### Western blot analyses

After being treated, the cardiomyocytes or cardiac tissues were homogenized in lysis buffer containing 50 mM Tris–HCl (pH 7.3), 150 mM NaCl, 5 mM EDTA, 1 mM dithiothreitol, 1% Triton X-100, and 1% protease inhibitor cocktail. The lysates were centrifuged for 15 min at 12,000 g, and the resulting supernatant was transferred to a new tube and stored at −70°C. The protein concentrations were determined using a Bradford protein assay kit, and the proteins were separated by electrophoresis and transferred to nitrocellulose membranes. The membranes were blocked for 1 h in Tris-buffered saline and Tween 20 (TBST, pH 7.6) that contained 5% non-fat dry milk and then incubated overnight at 4°C with antibodies against HIF-1α, iNOS(1∶500 dilution), Bcl2, Caspase3, or β-actin (1∶1,000 dilution) followed by washes with TBST. The membranes were then probed with appropriate secondary antibodies (1∶2,000 dilution) at room temperature for 90 min, followed by washes with TBST. The protein bands were detected by chemiluminescence and were quantified using the Quantity One software package (Bio-Rad Laboratories, UK).

### RNA silencing of HIF-1α

HIF-1α siRNA transfection was performed with siRNA transfection reagent according to the manufacturer's protocols. HIF-1α siRNA (2 nmol/L) was mixed with a transfection reagent added to primary neonatal cardiomyocytes and equivalent concentrations of scrambled sequence siRNA (con siRNA) were transfected for negative control. Silencing of HIF-1α transcription was confirmed by western blot.

### Statistical analysis

All of the experiments were performed in duplicate and repeated at least three times. The data are expressed as the means ± the standard error of the mean (SEM). Group comparisons were performed using an ANOVA (SPSS 13.0). All of the groups were analyzed simultaneously with a LSD t-test. A difference of P<0.05 was considered to be statistically significant.

## Results

### The effect of post-ischemia treatment with Ast IV on the viability of SIR-injured cardiomyocytes

**Figure 2:**
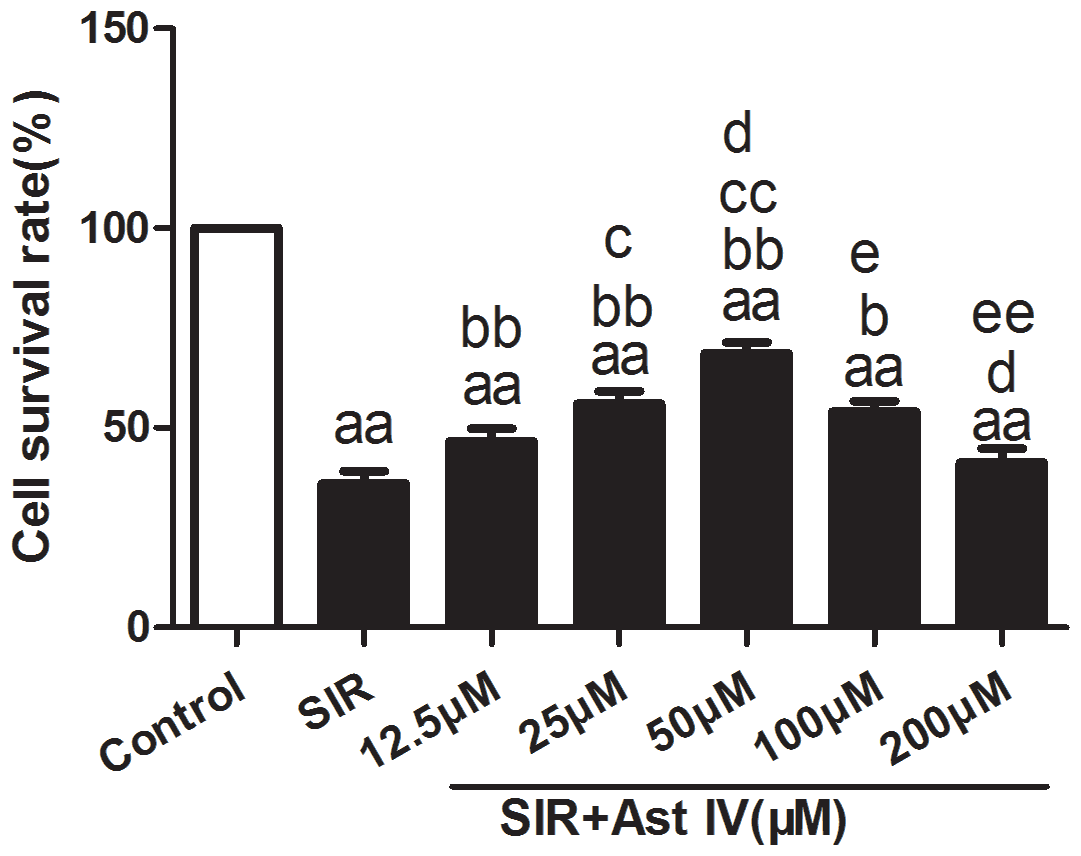
The effect of post-ischemia treatment with Ast IV on the viability of SIR-injured cardiomyocytes. Cardiomyocyte viability was assessed using the MTT assay. The results are expressed as the mean±SEM, n = 6. ^aa^P<0.01 vs. Control; ^b^P<0.05 vs. SIR; ^bb^P<0.01 vs. SIR; ^c^P<0.05 vs. SIR+Ast IV(12.5 µΜ); ^cc^P<0.01 vs. SIR+Ast IV(12.5 µΜ); ^d^P<0.05 vs. SIR+Ast IV(25 µΜ); ^e^P<0.05 vs. SIR+Ast IV(50 µΜ); ^ee^P<0.01 vs. SIR+Ast IV(50 µΜ). SIR, simulated ischemia reperfusion; Ast IV, Astragaloside IV.

Cultured cardiomyocyte survival following SIR was assessed by MTT assay (Figure 2). As expected, SIR induced significant decreases in cell viability to 18.7±1.9% compared with control cells (P<0.01), while Ast IV treatment resulted in a significant increase in cell survival, restoring the cell survival rate to 39.7±2.4% (12.5 µM), 44.1±3.1%(25 µM), 50.4±3.4%(50 µM), 41.7±2.1%(100 µM), and 19.2±1.7% (200 µM) (all P<0.01). The protective effect of 50 µM Ast IV was the most effective; therefore, this concentration was selected for further research. Additionally, the viability of the primary neonatal cardiomyocytes after 24 h, 48 h, or 72 h of treatment with Ast IV (12.5, 25, 50, 100, or 200 µM) was assessed using the MTT assay. As expected, Ast IV (12.5, 25, 50, or 100 µM) had no effect on the normal primary neonatal cardiomyocytes (Figure S1).

### The effects of Ast IV and 2-MeOE2 post-ischemia treatment on the viability, LDH release, and apoptotic index of SIR-injured cardiomyocytes

**Figure 3:**
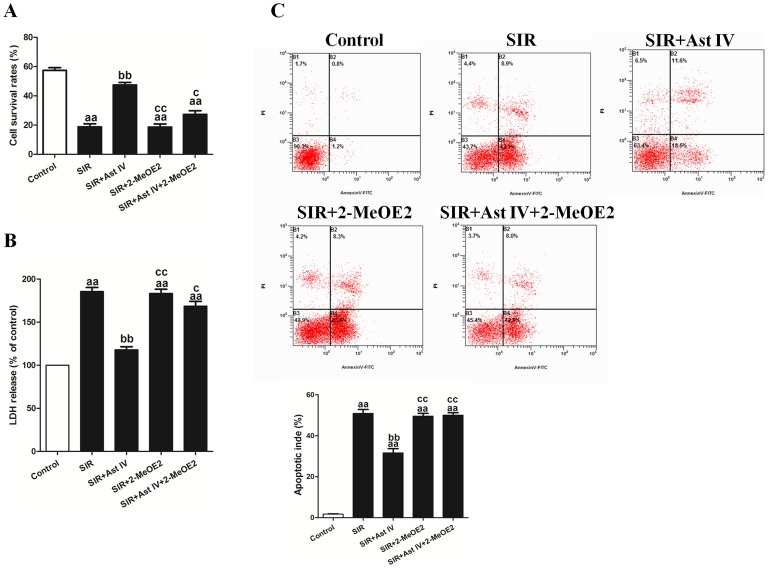
The effects of Ast IV and 2-MeOE2 post-ischemia treatment on cell viability, LDH release, and apoptotic index of SIR-injured cardiomyocytes. (A). Cardiomyocyte viability was assessed using the MTT assay. (B). An ELISA assay was performed to detect the release of LDH in culture medium. (C). Representative flow cytometry apoptotic results are shown. Four subpopulations and their fractions are indicated: normal cells (lower left), dead cells (upper left), early apoptotic cells (lower right), and late apoptotic cells (upper right). The apoptotic index is expressed as the number of apoptotic cells/the total number of counted cells ×100%. The results are expressed as the mean±SEM, n = 6. ^aa^P<0.01 vs. Control; ^bb^P<0.01 vs. SIR; ^cc^P<0.01 vs. SIR+Ast IV. SIR, simulated ischemia reperfusion; Ast IV, Astragaloside IV; 2-MeOE2, 2-methoxyestradiol; LDH, lactate dehydrogenase.

Post-ischemia treatment with Ast IV (50 µM) markedly increased the cell survival rate, to 50.4±3.4%, following SIR (vs. SIR group, P<0.01; Figure 3A). In addition, post-ischemia treatment with Ast IV significantly decreased the apoptotic index and LDH release (vs. SIR group, P<0.01; Figures 3B and 3C). In comparison with the control group, the Ast IV treatment group presented a non-significant effect on the cell survival rate and LDH release. However, the protective effect of post-ischemia treatment with Ast IV was abolished by 2-MeOE2 (vs. SIR+Ast IV group, P<0.01). Compared with the control treatment, SIR+Ast IV treatment did not have a significant impact on increasing the survival rate or decreasing the LDH release of the SIR-injured cardiomyocytes.

### The effects of Ast IV and 2-MeOE2 post-ischemia treatment on HIF-1α, iNOS, Bcl2, and Caspase3 expression in SIR-injured cardiomyocytes

As shown in Figure S2A, SIR+Ast IV-treatment significantly increased iNOS mRNA expression to 6.37±0.87-fold that of the control (vs. SIR group, P<0.01). However, this effect of SIR+Ast IV treatment on iNOS mRNA expression was abolished by 2-MeOE2 (vs. SIR+Ast IV+2-MeOE2 group, P<0.01). As expected, SIR+2-MeOE2 treatment significantly decreased iNOS expression, to 0.97±0.14-fold that of the control (vs. SIR group, P<0.01).

**Figure 4:**
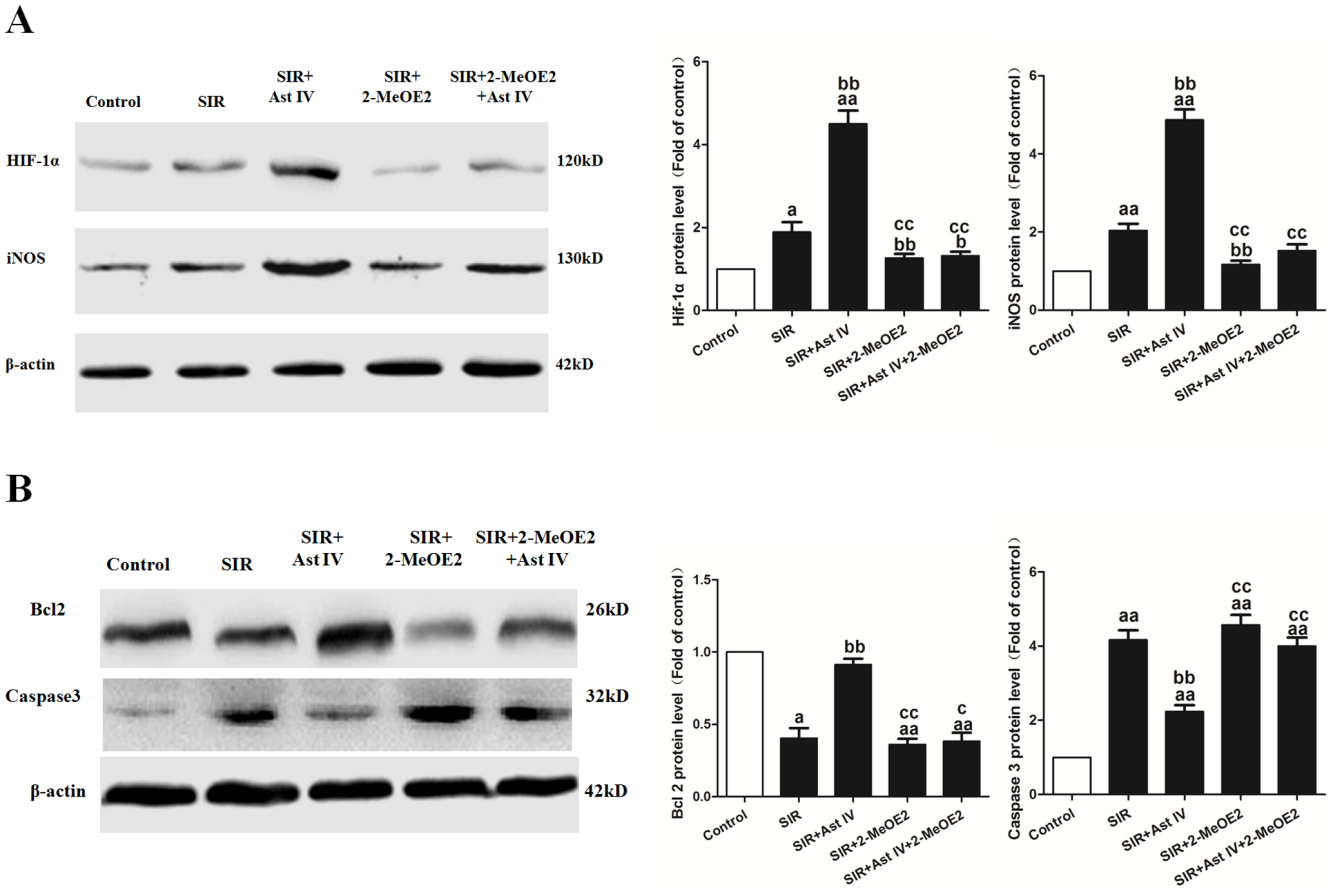
The effects of Ast IV and 2-MeOE2 post-ischemia treatment on HIF-1α, iNOS, Bcl2, and Caspase3 protein expression in SIR-injured cardiomyocytes. (A). Western blot image for HIF-1a and iNOS protein expression. (B). Western blot image for the expression of Bcl2 and Caspase3. The results are expressed as the mean±SEM, n = 6. ^a^P<0.05 vs. Control; ^aa^P<0.01 vs. Control; ^bb^P<0.01 vs. SIR; ^cc^P<0.01 vs. SIR+Ast IV. SIR, simulated ischemia reperfusion; Ast IV, Astragaloside IV; 2-MeOE2, 2-methoxyestradiol.

As shown in Figures 4A and 4B, post-ischemia treatment with Ast IV significantly increased HIF-1α, iNOS, and Bcl2 protein expression, to 4.36±0.20-fold, 4.11±0.14-fold, and 4.14±0.17-fold of the control, respectively, whereas Caspase3 expression decreased to 0.45±0.087-fold that of the control (vs. SIR group, P<0.01). The effects of post-ischemia treatment with Ast IV on the expression of HIF-1α, iNOS, Bcl2, and Caspase3 were abolished by 2-MeOE2 (vs. SIR+Ast IV group, P<0.01).

### The effects of Ast IV and 2-MeOE2 post-ischemia treatment on the cardiac function, infarct size, apoptotic index, and LDH release of IR-injured isolated hearts

**Figure 5:**
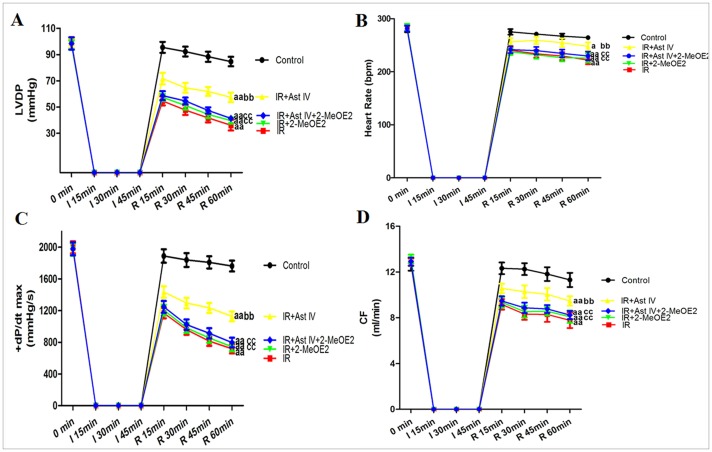
The effects of Ast IV and 2-MeOE2 post-ischemia treatment on cardiac function of IR-injured isolated hearts. (A). Representative line graph for the LVDP curve. (B). Representative line graph for the HR curve.(C). Representative line graph for the +dP/dt max curve. (D). Representative line graph for the CF curve. The results are expressed as the mean±SEM, n = 6. ^a^P<0.05 vs. Control; ^aa^P<0.01 vs. Control; ^b^P<0.05 vs. IR; ^bb^P<0.01 vs. IR; ^c^P<0.05 vs. IR+Ast IV; ^cc^P<0.01 vs. IR+Ast IV. IR, ischemia reperfusion; Ast IV, Astragaloside IV; 2-MeOE2, 2-methoxyestradiol; HR, heart rate; LVDP, left ventricular peak developing pressure; +dP/dt max, the maximum rate of pressure change in the ventricle; CF, coronary flow.

The results for the isolated rat hearts were consistent with those that were obtained for the cardiomyocytes. As expected, after the IR procedure, the absolute HR, LVDP, +dP/dt max, and coronary flow (CF) values were significantly decreased compared with the baseline values (P<0.01). Post-ischemia treatment with Ast IV significantly increased the functional recovery of the post-ischemic hearts, which was demonstrated by significantly higher HR, LVDP, +dP/dt max, and CF values throughout the reperfusion period compared with those of the IR group (P<0.01). Compared with the IR group, the IR+2-MeOE2 treatment had no significant influence on cardiac function (P>0.05). However, 2-MeOE2 significantly attenuated the functional recovery of the post-ischemic hearts, which was demonstrated by significantly lower HR, LVDP, +dP/dt max, and CF values throughout the reperfusion period compared with those of the IR+Ast IV group (P<0.01; Figures 5A–5D).

**Figure 6:**
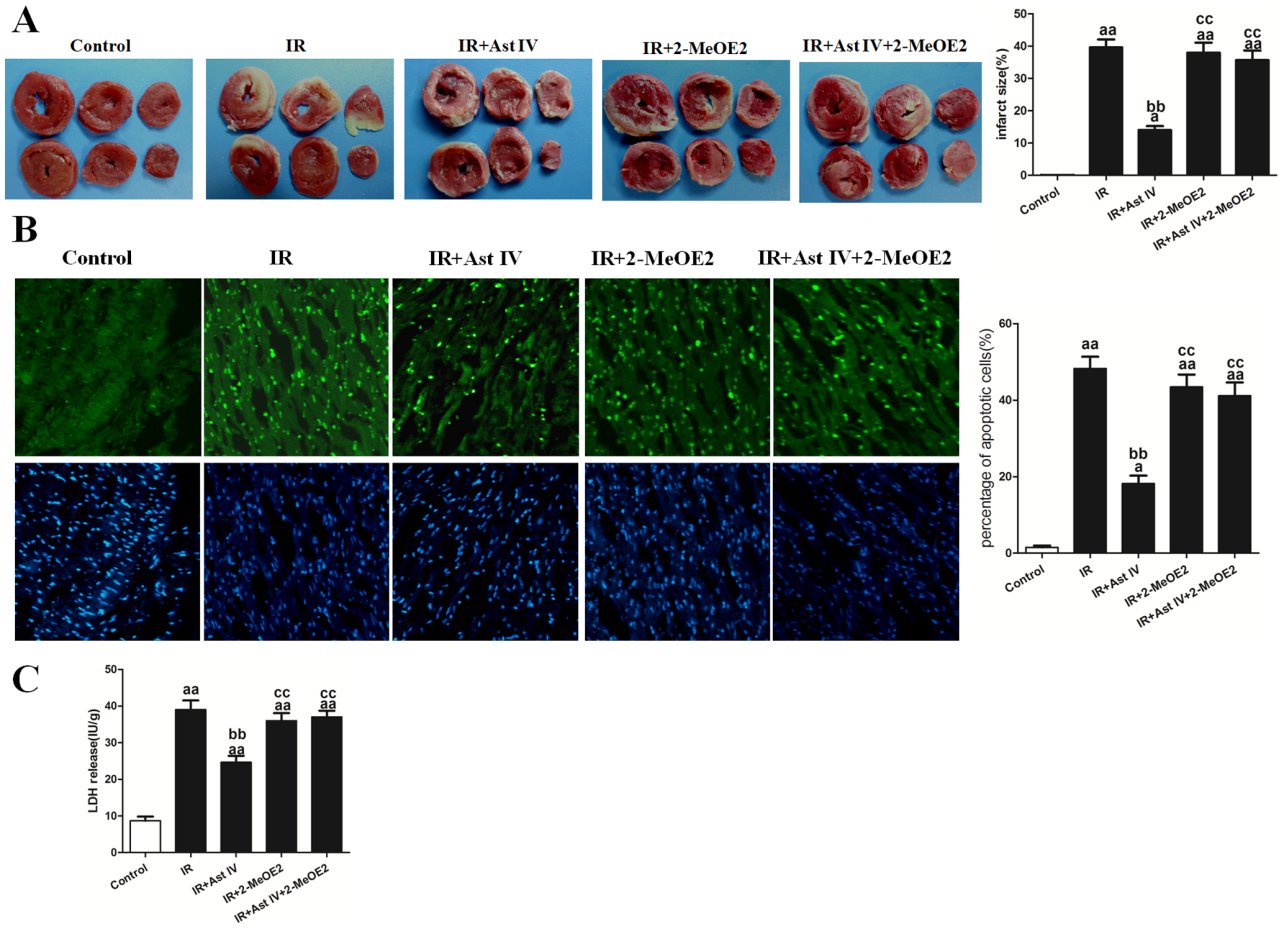
The effects of Ast IV and 2-MeOE2 post-ischemia treatment on the infarct size, apoptotic index, and LDH release of IR-injured isolated hearts. (A). Representative images of the myocardial infarct size are shown. The infarction size is expressed as the percentage of infarct relative to the mass at risk. (B). Representative images of apoptotic cardiomyocytes are shown. The apoptotic cells were detected by immunofluorescent staining with TUNEL (green)and DAPI (blue) staining was used to label the nuclei. (C). The amount of LDH was normalized against the dry weight of the heart and is expressed as IU/g. The results are expressed as the mean±SEM, n = 6. ^aa^P<0.01 vs. Control; ^bb^P<0.01 vs. IR; ^cc^P<0.01 vs. IR+Ast IV. IR, ischemia reperfusion; Ast IV, Astragaloside IV; 2-MeOE2, 2-methoxyestradiol; LDH, lactate dehydrogenase.

The infarct size (39.70±3.47%; Figure 6A) and the number of apoptotic cardiomyocytes (44.2±4.23%; Figure 6B) in the IR hearts were significantly reduced by post-ischemia treatment with Ast IV, to 13.50±2.07% and 18.28±2.46%, respectively (vs. IR group, P<0.01). In contrast, in the IR+Ast IV+2-MeOE2 group, the myocardial infarct size was 34.52±2.95%, and the percentage of apoptotic cardiomyocytes was 40.21±3.74% (vs. IR+Ast IV group, P<0.01). Compared with the IR treatment, IR+2-MeOE2 treatment had no significant influence on the myocardial infarct size or the number of apoptotic cardiomyocytes.

The total LDH release that was measured in the coronary effluent in the IR group was significantly increased compared with that in the control group (39.47±3.14 vs. 8.24±0.78 IU/g, P<0.01). Post-ischemia treatment with Ast IV significantly reduced LDH release in the IR+Ast IV group, to 25.17±2.84 IU/g (vs. IR group, P<0.01). Compared with IR treatment alone, IR+2-MeOE2 treatment had no significant influence on LDH release. However, compared with LDH release in the IR+Ast IV group, LDH release in the IR+Ast IV+2-MeOE2 group increased significantly, to 35.47±2.37 IU/g (vs. IR+Ast IV group, P<0.01; Figure 6C).

### The effects of post-ischemia treatment with Ast IV and 2-MeOE2 on HIF-1α, iNOS, Bcl2, and Caspase3 expression in IR-injured isolated hearts

As shown in Figure S2B, IR+Ast IV treatment significantly increased iNOS mRNA expression, to 5.48±0.65-fold that of the control (vs. IR group, P<0.01). However, the AST IV protective effects on iNOS mRNA expression was abolished by 2-MeOE2 (vs. IR+Ast IV+2-MeOE2 group, P<0.01). As expected, IR+2-MeOE2 treatment significantly decreased iNOS expression, to 1.24±0.17-fold that of the control (vs. IR group, P<0.01).

**Figure 7:**
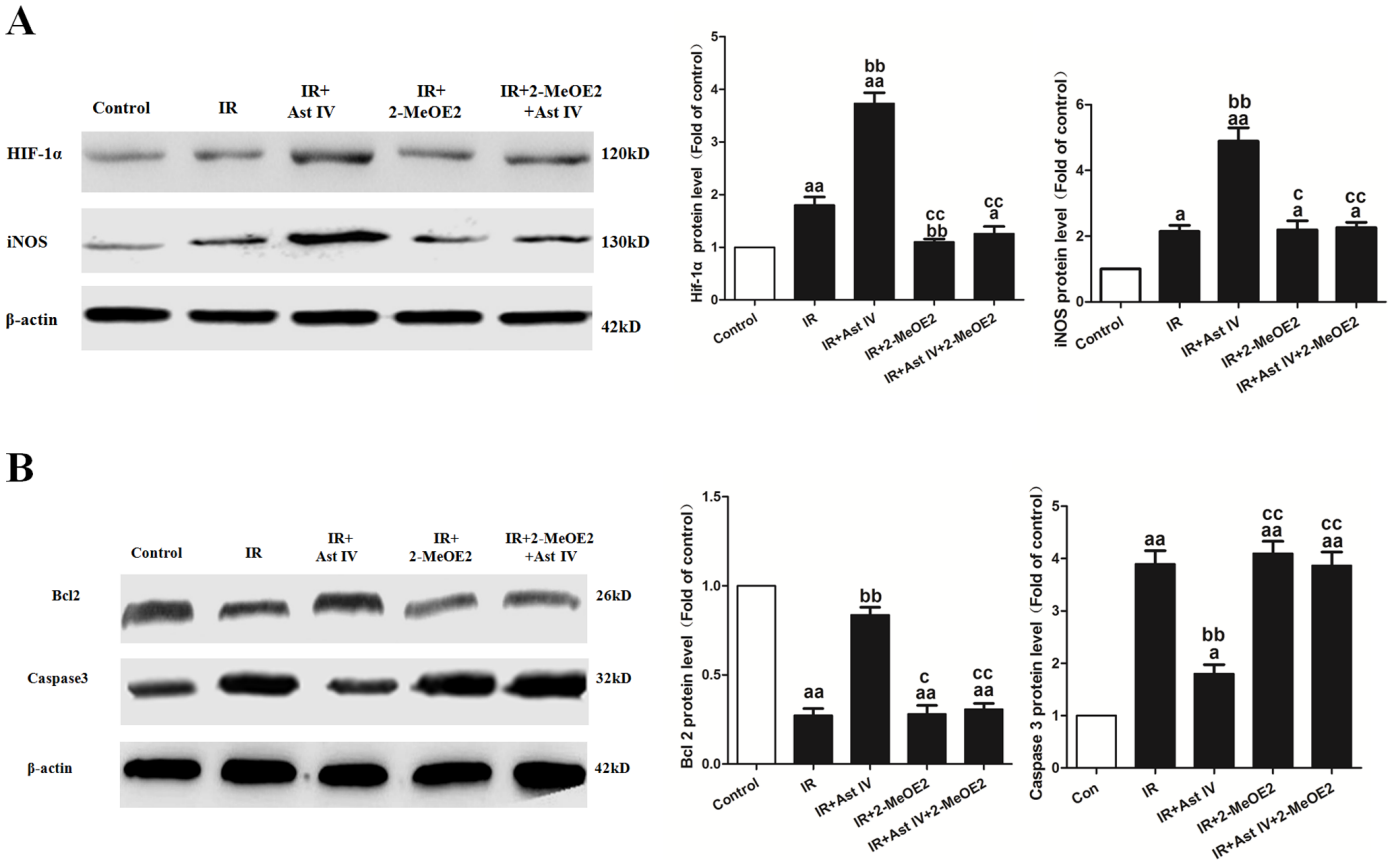
The effects of Ast IV and 2-MeOE2post-ischemia treatment on HIF-1α, iNOS, Bcl2, and Caspase3 protein expression in IR-injured isolated hearts. (A). Representative images of HIF-1α and iNOS expression are shown. (B). Representative images of Bcl2 and Caspase3 expression are shown. The results are expressed as the mean±SEM, n = 6. ^aa^P<0.01 vs. Control; ^bb^P<0.01 vs. IR; ^cc^P<0.01 vs. IR+Ast IV. IR, ischemia reperfusion; Ast IV, Astragaloside IV; 2-MeOE2, 2-methoxyestradiol.

As shown in Figures 7A and 7B, post-ischemia treatment with Ast IV significantly increased HIF-1α, iNOS, and Bcl2 protein expression, to 4.12±0.33-fold, 4.92±0.27-fold, and 0.82±0.033-fold that of the control, respectively, and decreased Caspase3 expression to 1.87±0.14-fold that of the control (vs. IR group, P<0.01). However, the AST IV protective effects on the protein expression of HIF-1α, iNOS, Bcl2, and Caspase3 were abolished by 2-MeOE2 (vs. IR+Ast IV group, P<0.01).

### The effects of Ast IV and HIF-1α siRNA post-ischemia treatment on SIRI cardiomyocytes

**Figure 8:**
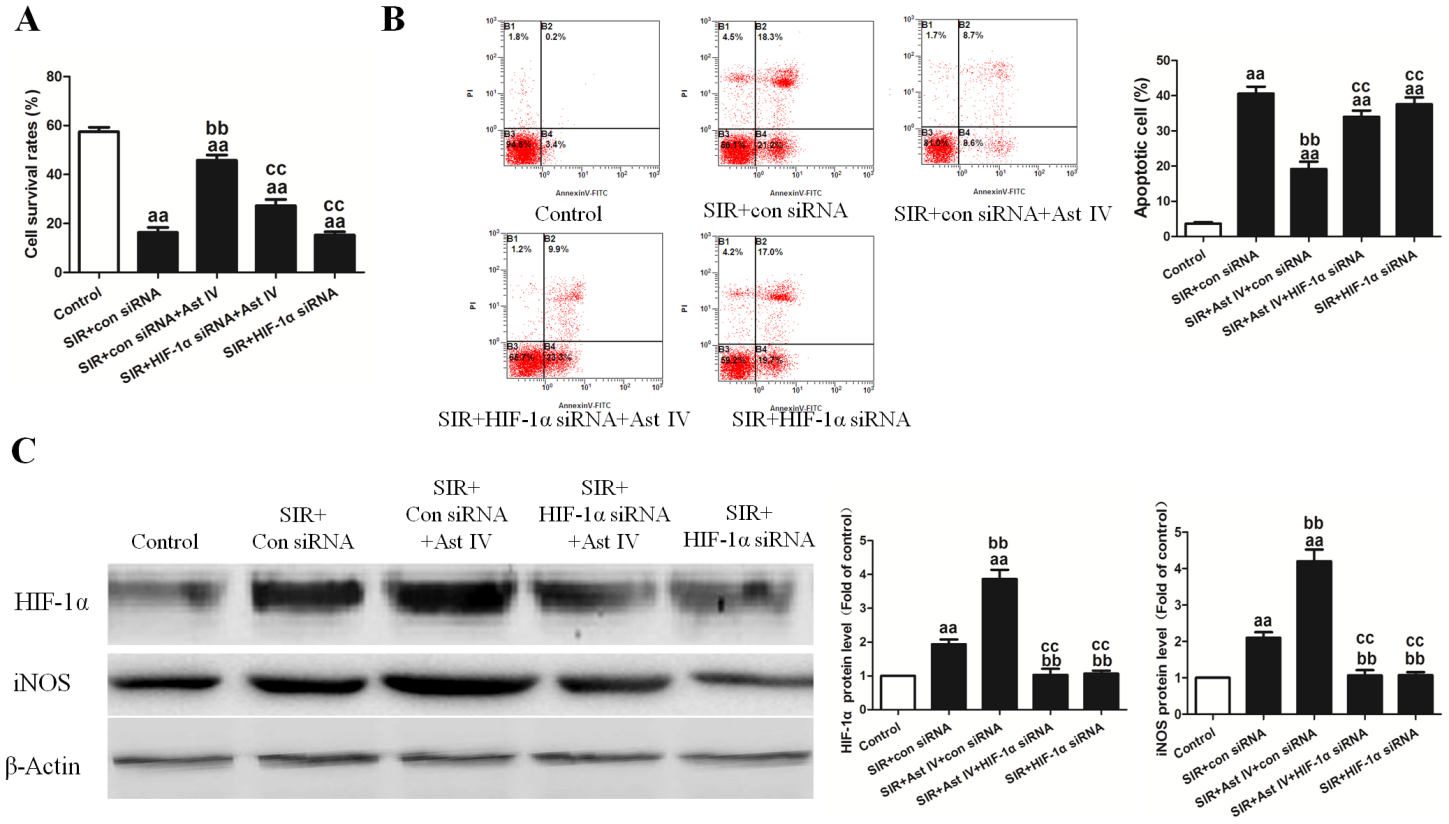
The effects of Ast IV and HIF-1α siRNA post-ischemia treatment on SIR-injured cardiomyocytes. (A) Cardiomyocyte viability was assessed using the MTT assay. (B) Representative flow cytometry apoptotic results are shown. Four subpopulations and their fractions are indicated: normal cells (lower left), dead cells (upper left), early apoptotic cells (lower right), and late apoptotic cells (upper right). The apoptotic index is expressed as the number of apoptotic cells/the total number of counted cells ×100%. (C) Representative images of HIF-1α and iNOS protein expression are shown. The results are expressed as the mean±SEM, n = 6. ^aa^P<0.01 vs. Control; ^bb^P<0.01 vs. SIR+Con siRNA; ^cc^P<0.01 vs. SIR+Con siRNA+Ast IV. ^dd^P<0.01 vs. SIR+HIF-1α siRNA+Ast IV. SIR, simulated ischemia reperfusion; Ast IV, Astragaloside IV.

Ast IV markedly increased the cell survival rate, to 48.7±5.1%, following SIR (vs. SIR group, P<0.01; Figure 8A). However, the AST IV protective effects as reflected by the increased cell survival rate were abolished by HIF-1α siRNA (vs. SIR+Ast IV+HIF-1α siRNA group, P<0.01; Figure 8A), not by control siRNA. Additionally, Ast IV treatment significantly decreased the apoptotic index (P<0.01; Figure 8B). However, the AST IV protective effects were abolished by HIF-1α siRNA (vs. SIR+Ast IV+HIF-1α siRNA group, P<0.01; Figure 8B), not by control siRNA.

As shown in Figure S2C, Ast IV treatment significantly increased iNOS mRNA expression, to 4.27±0.24-fold that of the control (vs. SIR+con siRNA group, P<0.01). However, the AST IV protective effects were abolished by HIF-1α siRNA (vs. SIR+Ast IV+HIF-1α siRNA group, P<0.01). As expected, SIR+HIF-1α siRNA treatment significantly decreased iNOS expression, to 1.14±0.18-fold that of the control (vs. SIR+con siRNA group, P<0.01).

As shown in Figure 8C, Ast IV treatment significantly increased HIF-1α and iNOS protein expression, to 3.66±0.54-fold and 4.41±0.21-fold that of the control, respectively (vs. SIR+con siRNA group, P<0.01). However, the AST IV protective effects were abolished by HIF-1α siRNA (vs. SIR+Ast IV+HIF-1α siRNA group, P<0.01)

## Discussion

To our knowledge, this is the first study to demonstrate that post-ischemia treatment with Ast IV conferred a cardioprotective effect on isolated rat hearts and cardiomyocytes, as evidenced by improved post-ischemic cardiac functional recovery (or cell viability), decreased myocardial infarct size, diminished LDH release into the coronary effluent (or cell culture medium), and a reduced number of apoptotic cardiomyocytes. In addition, the cardioprotective effect of Ast IV treatment was abolished by 2-MeOE2 or HIF-1α siRNA. These results indicate that the cardioprotective effect of Ast IV treatment is at least partially dependent on activation of the HIF-1α signaling pathway.

Ast IV, which possesses a variety of pharmacological and biological properties, can potentially mediate cardiovascular protection [35]. This compound's protective effects against myocardial IRI have been reported in different animal models. For example, Ast IV prevents IR-induced cardiac malfunction and maintains the integrity of the myocardial structure by regulating energy metabolism. Aside from its cardioprotective effects, 20 mg/L Ast IV treatment shows no adverse effects on normal perfused hearts [16]. Our results further demonstrated that post-ischemia treatment with Ast IV mediated significant protective effects against IRI.

Originally described as a master regulator of oxygen homeostasis, the HIF-1α pathway has been recognized as an important signaling pathway for a variety of stress responses, including ischemia, hypoxia, and oxidative stress [18]. The HIF-1α signaling pathway has also been recognized as a fundamental component of the intrinsic survival pathways that protect against IRI [20]–[23]. A recent study revealed that Ast IV is a novel regulator of HIF-1α and angiogenesis through the PI3K/Akt pathway in human umbilical vein endothelial cells (HUVECs) that are exposed to hypoxia [24]. 2-MeOE2, an endogenous metabolite of estradiol, inhibits HIF-1α and is an anti-angiogenic and antitumor agent [36]. Recently published reports have provided in vitro and in vivo evidence that 2-MeOE2 has a direct effect on HIF-1α inhibition and is not the result of a “side effect” of mitotic arrest [37], [38]. In addition, at moderate concentrations, 2-MeOE2 has no direct toxic effects on slowly dividing non-tumor cells; however, it does lead to reliable inhibition of HIF-1α [36]. Based on our preliminary experiments, 5 µM 2-MeOE2-treated cardiomyocytes and 2 µM 2-MeOE2-treated isolated hearts were used to investigate the role of the HIF-1α pathway in mediating the protective effects of post-ischemia treatment with Ast IV against myocardial IRI. Our results showed that 2-MeOE2 treatment led to loss of the protection conferred by Ast IV, and this pharmacological inhibition of HIF-1α signaling also abolished the Ast IV-induced increase in Bcl2 and decrease in Caspase3, which strongly suggested a possible interaction of HIF-1α and the anti-apoptotic pathways.

In the cardiomyocyte model, two strategies were used to explore the role of HIF-1α in the protective effects of post-ischemia treatment with Ast IV. First, the cardiomyocytes were exposed to Ast IV during the reperfusion procedure in the presence or absence of incubation with the HIF-1α inhibitor 2-MeOE2. Second, the cardiomyocytes were exposed to Ast IV during the reperfusion procedure with or without transfection with HIF-1α siRNA. The results confirmed that post-ischemia treatment with Ast IV can attenuate SIRI via HIF-1α activation, which transmits a survival signal to the myocardium.

NO, as a regulator of diverse pathophysiological mechanisms, plays an important role in the cardiovascular system. When NO is synthesized in large quantities by activated cells, it has cytotoxic properties, and it has been implicated in the pathophysiology of cardiovascular diseases [39]. Among the at least three isoforms of NOS, iNOS is upregulated by hypoxia in cardiomyocytes [40]. iNOS is possibly induced by HIF-1α [41]. iNOS-NO induction may partially protect cardiomyocytes from SIRI. Belaidi and colleagues found that HIF-1α and iNOS appear to play a pivotal role in the delayed pharmacological myocardial preconditioning induced by cobalt, thus mimicking the effects of hypoxic preconditioning [42]. In the present study, the upregulation of iNOS by post-ischemia treatment with Ast IV was reversed by 2-MeOE2 treatment, which indicates that HIF-1α activation by Ast IV may result in the activation of iNOS signaling and confer protection against myocardial IRI.

In summary, post-ischemia treatment with Ast IV can attenuate myocardial IRI through activating HIF-1α/iNOS pathway, which transmits a survival signal to the myocardium. In most circumstances, clinicians must save the ischemic myocardium following acute myocardial infarction or after unexpected myocardial stunning following a significant period of ischemic heart arrest during complicated heart surgery, which means that a post-ischemia treatment method of cardioprotection maybe more important than a pre-treatment method in clinical settings. Our results indicate that the attractiveness of post-ischemia treatment with Ast IV was due to its protective stimulus, which could be introduced at reperfusion; thus, post-ischemia treatment with Ast IV is more predictable and is potentially feasible in the clinical setting.

## Supporting Information

Figure S1 **The effect of Ast IV on the cell viability of normal cardiomyocytes.** The results are expressed as the mean±SEM, n = 6. ^a^P<0.05 vs. Control; ^aa^P<0.01 vs. Control.
(TIF)

Figure S2 **iNOS mRNA expression before and after all sets of treatments.** (A) Real time RT-PCR for evaluating iNOS mRNA expression of SIR-injured cardiomyocytes, and β-actin was used as internal control. The results are expressed as the mean±SEM, n = 6. ^aa^P<0.01 vs. Control; ^b^P<0.05 vs. SIR; ^bb^P<0.01 vs. SIR. ^cc^P<0.01 vs. SIR+Ast IV. SIR, simulated ischemia reperfusion; Ast IV, Astragaloside IV.(B). Real time RT-PCR for evaluating iNOS mRNA expression of IR-injured isolated hearts, and β-actin was used as internal control. The results are expressed as the mean±SEM, n = 6. ^aa^P<0.01 vs. Control; ^bb^P<0.01 vs. IR; ^cc^P<0.01 vs. IR+Ast IV. IR, ischemia reperfusion; Ast IV, Astragaloside IV.(C). Real time RT-PCR for evaluating iNOS mRNA expression of SIR-injured cardiomyocytes, and β-actin was used as internal control. The results are expressed as the mean±SEM, n = 6. ^aa^P<0.01 vs. Control; ^bb^P<0.01 vs. SIR+con siRNA. ^cc^P<0.01 vs. SIR+con siRNA+Ast IV. SIR, simulated ischemia reperfusion; Ast IV, Astragaloside IV.
(TIF)

## References

[1] Hausenloy DJ, Boston-Griffiths E, Yellon DM (2012) Cardioprotection during cardiac surgery. Cardiovasc Res 94(2): 253–265. 22440888 10.1093/cvr/cvs131 PMC3331616

[2] Yellon DM, Hausenloy DJ (2007) Myocardial reperfusion injury. N Engl J Med 357(23): 2408.

[3] Schwartz Longacre L, Kloner RA, Arai AE, Baines CP, Bolli R, et al (2011) New horizons in cardioprotection: recommendations from the 2010 National Heart, Lung, and Blood Institute Workshop. Circulation 124(10): 1172–1179. 21900096 10.1161/CIRCULATIONAHA.111.032698 PMC3709973

[4] Ovize M, Baxter GF, Di Lisa F, Ferdinandy P, Garcia-Dorado D, et al (2010) Postconditioning and protection from reperfusion injury: where do we stand? Position paper from the Working Group of Cellular Biology of the Heart of the European Society of Cardiology. Cardiovasc Res 87(3): 406–423. 20448097 10.1093/cvr/cvq129

[5] Mewton N, Bochaton T, Ovize M (2013) Postconditioning the heart of ST-elevation myocardial infarction patients. Circ J 77(5): 1123–1130. 23575305 10.1253/circj.cj-13-0385

[6] Skyschally A, van Caster P, Iliodromitis EK, Schulz R, Kremastinos DT, et al (2009) Ischemic postconditioning: experimental models and protocol algorithms. Basic Res Cardiol 104(5): 469–483. 19543787 10.1007/s00395-009-0040-4

[7] Hausenloy DJ, Baxter G, Bell R, Bøtker HE, Davidson SM, et al (2010) Translating novel strategies for cardioprotection: the Hatter Workshop Recommendations. Basic Res Cardiol 105(6): 677–686. 20865418 10.1007/s00395-010-0121-4 PMC2965360

[8] Eltzschig HK, Eckle T (2011) Ischemia and reperfusion-from mechanism to translation. Nature medicine 17(11): 1391–1401. 10.1038/nm.2507 PMC3886192 22064429

[9] Ren S, Zhang H, Mu Y, Sun M, Liu P (2013) Pharmacological effects of Astragaloside IV: a literature review. J Tradit Chin Med 33(3): 413–416. 24024343 10.1016/s0254-6272(13)60189-2

[10] Li M, Qu YZ, Zhao ZW, Wu SX, Liu YY, et al (2012) Astragaloside IV protects against focal cerebral ischemia/reperfusion injury correlating to suppression of neutrophils adhesion-related molecules. Neurochem Int 60(5): 458–465. 22342823 10.1016/j.neuint.2012.01.026

[11] Gui D, Huang J, Liu W, Guo Y, Xiao W, et al (2013) Astragaloside IV prevents acute kidney injury in two rodent models by inhibiting oxidative stress and apoptosis pathways. Apoptosis 18(4): 409–422. 23325448 10.1007/s10495-013-0801-2

[12] Cheng MX, Chen ZZ, Cai YL, Liu CA, Tu B (2011) Astragaloside IV Protects Against Ischemia Reperfusion in a Murine Model of Orthotopic Liver Transplantation. Transplantat Proc 43(5): 1456–1461. 10.1016/j.transproceed.2011.02.066 21693217

[13] Gui D, Huang J, Guo Y, Chen J, Chen Y, et al (2013) Astragaloside IV ameliorates renal injury in streptozotocin-induced diabetic rats through inhibiting NF-κB-mediated inflammatory genes expression. Cytokine 61(3): 970–977. 23434274 10.1016/j.cyto.2013.01.008

[14] Chen X, Peng LH, Li N, Li QM, Li P, et al (2012) The healing and anti-scar effects of astragaloside IV on the wound repair in vitro and in vivo. J Ethnopharmacol 139(3): 721–727. 22143155 10.1016/j.jep.2011.11.035

[15] Li ZP, Cao Q (2002) Effects of astragaloside IV on myocardial calcium transport and cardiac function in ischemic rats. Acta Pharmacol Sin 23(10): 898–904. 12370095

[16] Zhang WD, Chen H, Zhang C, Liu RH, Li HL, et al (2006) Astragaloside IV from Astragalus membranaceus shows cardioprotection during myocardial ischemia in vivo and in vitro. Planta Med 72(1): 4–8. 16450288 10.1055/s-2005-873126

[17] Wu X, Cao Y, Nie J, Liu H, Lu S, et al (2013) Genetic and Pharmacological Inhibition of Rheb1-mTORC1 Signaling Exerts Cardioprotection against Adverse Cardiac Remodeling in Mice. The American Journal of Pathology 182(6): 2005–2014. 23567640 10.1016/j.ajpath.2013.02.012

[18] Semenza GL (2012) Hypoxia-inducible factors in physiology and medicine. Cell 148(3): 399–408. 22304911 10.1016/j.cell.2012.01.021 PMC3437543

[19] Lee SH, Wolf PL, Escudero R, Deutsch R, Jamieson SW, et al (2000) Early expression of angiogenesis factors in acute myocardial ischemia and infarction. N Engl J Med 342(9): 626–633. 10699162 10.1056/NEJM200003023420904

[20] Adluri RS, Thirunavukkarasu M, Dunna NR, Zhan L, Oriowo B, et al (2011) Disruption of Hypoxia-Inducible Transcription Factor-Prolyl Hydroxylase Domain-1 (PHD-1-/-) Attenuates Ex Vivo Myocardial Ischemia/Reperfusion Injury Through Hypoxia-Inducible Factor-1 a Transcription Factor and Its Target Genes in Mice. Antioxid & redox signal 15(7): 1789–1797. 10.1089/ars.2010.3769 PMC3159109 21083501

[21] Cai Z, Zhong H, Bosch-Marce M, Fox-Talbot K, Wang L, et al (2008) Complete loss of ischaemic preconditioning-induced cardioprotection in mice with partial deficiency of HIF-1 a. Cardiovasc Res 77(3): 463–470. 18006459 10.1093/cvr/cvm035

[22] Zhao HX, Wang XL, Wang YH, Wu Y, Li XY, et al (2010) Attenuation of myocardial injury by postconditioning: role of hypoxia inducible factor-1 a. Basic Res Cardiol 105(1): 109–118. 19597757 10.1007/s00395-009-0044-0

[23] Fang Li Q, Xu H, Sun Y, Hu R, Jiang H (2012) Induction of inducible nitric oxide synthase by isoflurane post-conditioning via hypoxia inducible factor-1αduring tolerance against ischemic neuronal injury. Brain Res 1451: 1–9. 22445062 10.1016/j.brainres.2012.02.055

[24] Zhang L, Liu Q, Lu L, Zhao X, Gao X, et al (2011) Astragaloside IV Stimulates Angiogenesis and Increases Hypoxia-Inducible Factor-1 Accumulation via Phosphatidylinositol 3-Kinase/Akt Pathway. J Pharmacol Exp Ther 338(2): 485–491. 21576377 10.1124/jpet.111.180992

[25] Hellwig-Burgel T, Stiehl DP, Wagner AE, Metzen E, Jelkmann W (2005) hypoxia-inducible factor-1 (HIF-1): a novel transcription factor in immune reactions. J Interferon Cytokine Res 25: 297–310. 15957953 10.1089/jir.2005.25.297

[26] Cuong DV, Kim N, Youm JB, Joo H, Warda M, et al (2006) Nitric oxide-cGMP- protein kinase G signaling pathway induces anoxic preconditioning through activation of ATP-sensitive K+channels in rat hearts. Am J Physiol Heart Circ Physiol 290(5): H1808–17. 16339835 10.1152/ajpheart.00772.2005

[27] Heusch G, Boengler K, Schulz R (2008) Cardioprotection: nitric oxide, protein kinases, and mitochondria. Circulation 118(19): 1915–1919. 18981312 10.1161/CIRCULATIONAHA.108.805242

[28] Jones SP, Bolli R (2006) The ubiquitous role of nitric oxide in cardioprotection. J Mol Cell Cardiol 40(1): 16–23. 16288777 10.1016/j.yjmcc.2005.09.011

[29] Brune B, Zhou J (2007) Nitric oxide and superoxide: interference with hypoxic signaling. Cardiovasc Res 75(2): 275–282. 17412315 10.1016/j.cardiores.2007.03.005

[30] Yang Y, Duan W, Lin Y, Yi W, Liang Z, et al (2013) SIRT1 activation by curcumin pretreatment attenuates mitochondrial oxidative damage induced by myocardial ischemia reperfusion injury. Free Radic Biol Med 65C: 667–679. 10.1016/j.freeradbiomed.2013.07.007 23880291

[31] Smart N, Mojet MH, Latchman DS, Marber MS, Duchen MR, et al (2006) IL-6 induces PI 3-kinase and nitric oxide-dependent protection and preserves mitochondrial function in cardiomyocytes. Cardiovascular Research 69(1): 164–177. 16219301 10.1016/j.cardiores.2005.08.017

[32] Punn A, Mockridge JW, Farooqui S, Marber MS, Heads RJ (2000) Sustained activation of p42/p44 mitogen-activated protein kinase during recovery from simulated ischemia mediates adaptive cytoprotection in cardiomyocytes. Biochem J. Pt 3: 891–9. PMC1221324 10970806

[33] Esumi K, Nishida M, Shaw D, Smith TW, Marsh JD (1991) NADH measurements in adult rat myocytes during simulated ischemia. Am J Physiol. 260(6 Pt 2): H1743–52. 10.1152/ajpheart.1991.260.6.H1743 2058713

[34] Yang Y, Duan W, Jin Z, Yi W, Yan J, et al (2013) JAK2/STAT3 activation by melatonin attenuates the mitochondrial oxidative damage induced by myocardial ischemia/reperfusion injury. J Pineal Res 55(3): 275–286. 23796350 10.1111/jpi.12070

[35] Zhao J, Yang P, Li F, Tao L, Ding H, et al (2012) Therapeutic effects of astragaloside IV on myocardial injuries: multi-target identification and network analysis. PLoS One 7(9): e44938. 23028693 10.1371/journal.pone.0044938 PMC3444501

[36] Mabjeesh NJ, Escuin D, LaVallee TM, Pribluda VS, Swartz GM, et al (2003) 2ME2 inhibits tumor growth and angiogenesis by disrupting microtubules and dysregulating HIF. Cancer Cell 3(4): 363–375. 12726862 10.1016/s1535-6108(03)00077-1

[37] Salama SA, Kamel MW, Botting S, Salih SM, Borahay MA (2009) Catechol-o-methyltransferase expression and 2-methoxyestradiol affect micro-toubule dynamics and modify steroid receptor signaling in leiomyoma cells. PLOS One 4(10): e7356. 19809499 10.1371/journal.pone.0007356 PMC2752809

[38] Kim SR, Lee KS, Park HS, Park SJ, Min KH, et al (2010) HIF-1alpha inhibition ameliorates an allergic airway disease via VEGF suppression in bronchial epithelium. Eur J Immunol 40(10): 2858–69. 20827786 10.1002/eji.200939948

[39] Lei J, Vodovotz Y, Tzeng E, Billiar TR (2013) Nitric Oxide, A Protective Molecule in the Cardiovascular System. Nitric Oxide 35C: 175–185. 10.1016/j.niox.2013.09.004 24095696

[40] Strijdom H, Friedrich SO, Hattingh S, Chamane N, Lochner A (2009) Hypoxia-induced regulation of nitric oxide synthase in cardiac endothelial cells and myocytes and the role of the PI3-K/PKB pathway. Mol Cell Biochem 321(1–2): 23–35. 18791856 10.1007/s11010-008-9906-2

[41] Dijkers PF, O'Farrell PH (2009) Dissection of a hypoxia-induced, nitric oxide-mediated signaling cascade. Mol Biol Cell 20(18): 4083–4090. 19625446 10.1091/mbc.E09-05-0362 PMC2743626

[42] Belaidi E, Beguin PC, Levy P, Ribuot C, Godin-Ribuot D (2012) Delayed myocardial preconditioning induced by cobalt chloride in the rat: HIF-1α and iNOS involvement. Fundam Clin Pharmacol 26(4): 454–462. 21480981 10.1111/j.1472-8206.2011.00940.x

