# Supplementary figures and images for: HIF-1α Signaling Activation by Post-Ischemia Treatment with Astragaloside IV Attenuates Myocardial Ischemia-Reperfusion Injury
Source: PLoS One. 2014 Sep 19;9(9):e107832. doi: 10.1371/journal.pone.0107832 (PMC4169594; doi:10.1371/journal.pone.0107832)

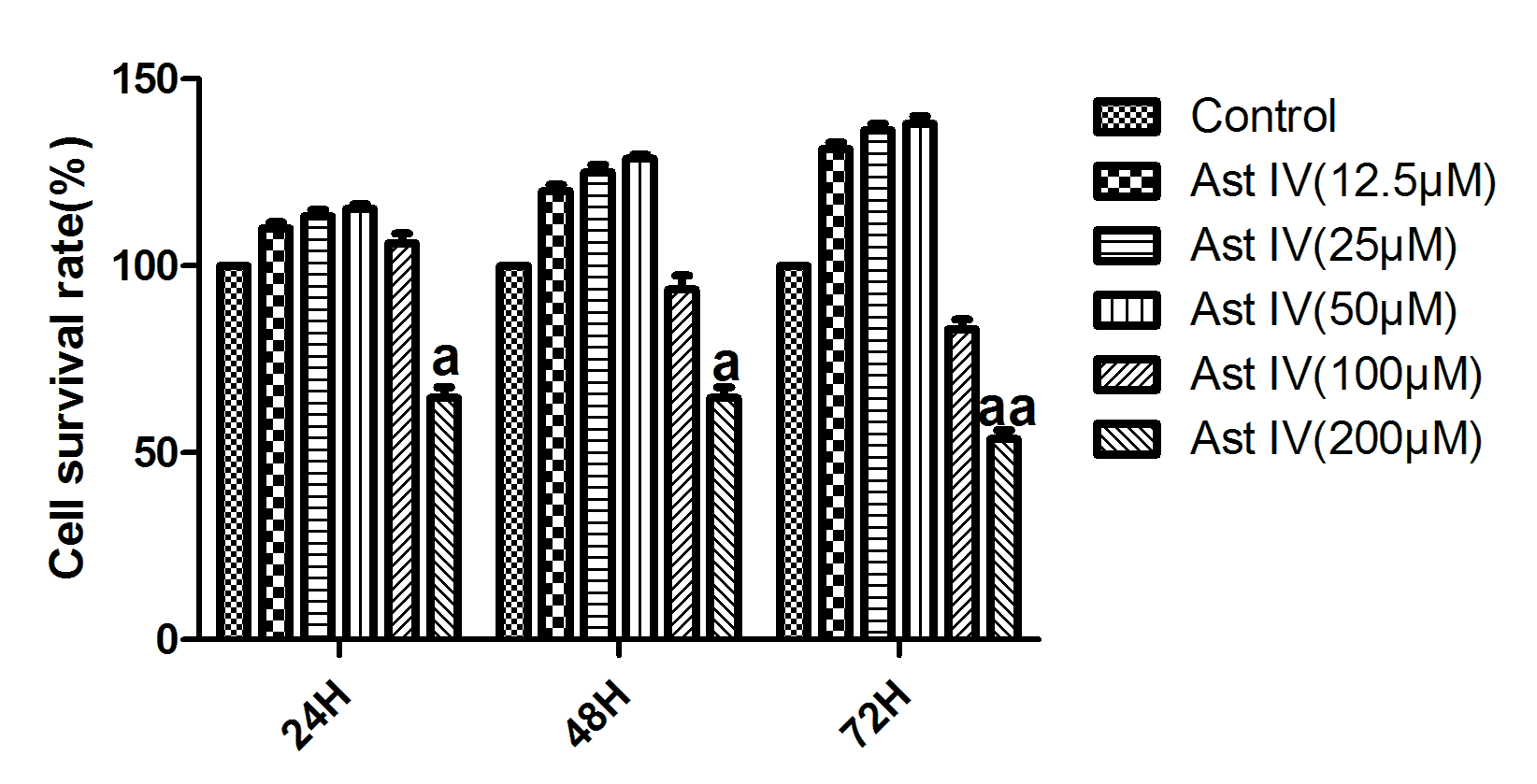

Supplement: Figure S1 — The effect of Ast IV on the cell viability of normal cardiomyocytes. The results are expressed as the mean±SEM, n = 6. aP<0.05 vs. Control; aaP<0.01 vs. Control. (TIF) [file pone.0107832.s001.tif]

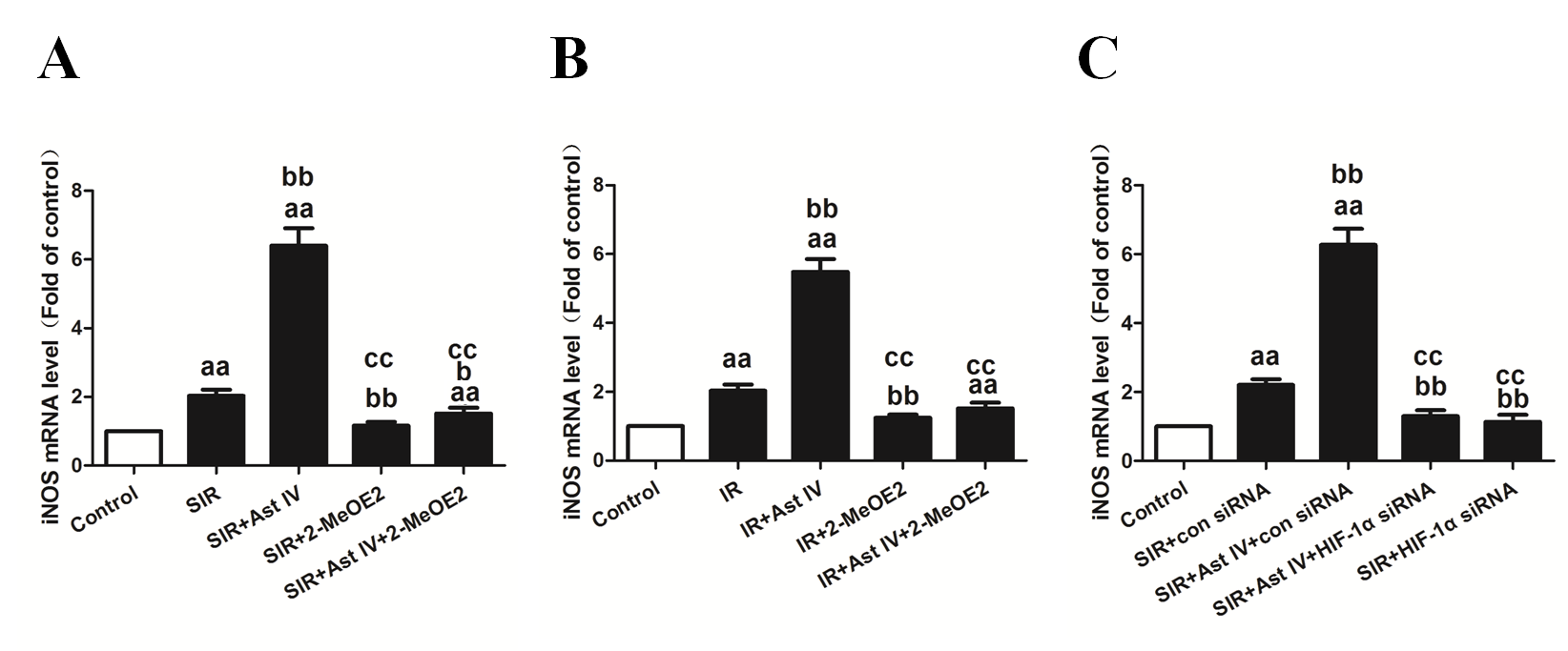

Supplement: Figure S2 — iNOS mRNA expression before and after all sets of treatments. (A) Real time RT-PCR for evaluating iNOS mRNA expression of SIR-injured cardiomyocytes, and β-actin was used as internal control. The results are expressed as the mean±SEM, n = 6. aaP<0.01 vs. Control; bP<0.05 vs. SIR; bbP<0.01 vs. SIR. ccP<0.01 vs. SIR+Ast IV. SIR, simulated ischemia reperfusion; Ast IV, Astragaloside IV.(B). Real time RT-PCR for evaluating iNOS mRNA expression of IR-injured isolated hearts, and β-actin was used as internal control. The results are expressed as the mean±SEM, n = 6. aaP<0.01 vs. Control; bbP<0.01 vs. IR; ccP<0.01 vs. IR+Ast IV. IR, ischemia reperfusion; Ast IV, Astragaloside IV.(C). Real time RT-PCR for evaluating iNOS mRNA expression of SIR-injured cardiomyocytes, and β-actin was used as internal control. The results are expressed as the mean±SEM, n = 6. aaP<0.01 vs. Control; bbP<0.01 vs. SIR+con siRNA. ccP<0.01 vs. SIR+con siRNA+Ast IV. SIR, simulated ischemia reperfusion; Ast IV, Astragaloside IV. (TIF) [file pone.0107832.s002.tif]
